# Work Life Balance – What Balance?

**DOI:** 10.3389/fped.2015.00102

**Published:** 2016-01-08

**Authors:** Tricia Shinelle Alleyne

**Affiliations:** ^1^Department of Pediatrics, Division of Critical Care, University of Tennessee Health Sciences Center, Memphis, TN, USA

**Keywords:** work, life balance, ephemeral, essential, time management

“*Never get so busy making a living that you forget to make a life*.” Anonymous. This quote stands out because as we highlight our career goals and strategically plan for them, our personal lives often happen as an afterthought. To be successful at achieving a work–life balance, we should pay equal attention to planning all spheres of our life. While God and Family have been the bedrock in my own life, I am still being groomed for a successful career. On closer examination, I have found that I was subtly prepared for personal life as well. I still remember my Aunt calling me with the following words, “Come and learn to cook so you won’t starve.” Or “Come and learn to do laundry so you can wear clean clothes.”

While I made clear career goals and worked methodically toward them, my personal life was sacrificed on the altar of success, with the expectation that someday I would pay myself back. That strategy works well in the short-term, but in the long run, it breeds resentment, bitterness, and an unbalanced life and dare I say it is unsafe. It took tragedy of unprecedented proportions to teach me that in the game of life there are no opportunities for payback. Shanafelt et al. in their paper “Burnout and satisfaction with work life balance among us physicians relative to the general US population” found that physicians were more likely to be dissatisfied with their work–life balance compared to the general population (40.2 vs. 23.2%). Additionally, physicians were at a higher risk of burnout 37.9 vs. 27.8% (*p* < 0.001) (1). The same authors found, 40% of physicians reported burnout, of those emergency medicine physicians were most likely to report burnout. In a separate observational cohort study performed by Garcia, which compared general pediatricians to pediatric intensivist, they found 71% of intensivists vs. 29% of general pediatricians were burnout (*p* < 0.01). (2). Hence, a lack of balance can lead to emotional exhaustion, depersonalization of our patients and their families, and lack of professional accomplishments (2).

In the business of life, the journey is as important as the destination. Time does not stop so you can achieve a goal, and our responsibility to our families does not pause for our careers. No one ever says, “I wish that I’d spent more time at work” on their deathbed. What good is it that we are internationally known for our career and our family only knows our name? Or we have achieved the ideal family life but our patients suffer a negative outcome because their doctor was rushing to punch the clock. Dare I say that you have not achieved success if we succeed in one area of your life and the rest of our life is in shambles? Another study found the presence of work–home conflicts in the past 3 weeks, and resolution of that conflict in favor of work was among the top three contributors to burnout (3).

I achieved a work–life balance by understanding and defining my priorities – I value my faith and my family above my career, my career above leisure, and leisure above wealth. I may not always be allowed to structure my time in complete harmony with these principles, but they all seem to even out. Now, while I cannot attend every family function, I make sure to attend the ones I should not miss; I may not snag the highest accolades but I attain the ones that carry the most value to me. I love my job, but it does not steal from my family and in turn, my family does not steal from job. There are days when emergencies occur and contrary to popular belief, there are systems in place to accommodate. It is those systems that ensure my balance – my husband supports me when I have to stay late or when I need to study or work on my fellowship research project. Oppositely, when I have family emergencies, my colleagues make it possible for me to respond to a family emergency seamlessly.

I am still learning how to say no to detractors and other’s expectations. While it looks good to be on a committee, I cannot be on every committee. I only have set time on this earth to achieve my purpose, and the challenge is to define and complete it in the allotted time. For example, on most clinical days, I am too tired to go home and read about an interesting patient but I have gotten around that by reading 1 h prior to starting my work day, on my downtime at work, reading a quick topic before I leave at the end of the day, instead of being unproductive.

Mayo Clinic Proceedings polled 900 spouses of physicians; they found that the strongest predictor of relationship satisfaction was the amount of awake time spent with their spouse daily. When I am night service, there are times I will not see my husband for a week but we talk at least once a day. Carol Ascherener, AAMC Chief Medical Education Officer, stated that achieving work–life balance is determined by how you spend your “discretionary time” (4). As a Fellow, “discretionary time” is defined by a finite period of time; so it is okay to unplug your devices, delegate the mundane tasks, and “plug in” to yourself (5, 6), your family, or your education.

Undoubtedly, as our careers surrounded by circumstances and responsibilities change, our priorities adjust themselves accordingly. Our ability to be flexible (in our thinking and schedules) and resourceful yet masterful in problem solving will be invaluable in maintaining a balanced life. My life is balanced by a thought that I love my family with all my heart, and I must give to my patients what I want for my family.

For each person, I challenge you to assign your priorities and schedule your time in accordance with them (7). Never forget what you do matters. I will end this article in the words of Dr. Bill Gentry, “You aren’t perfect. All you can be is your best. Be your best at work. Be your best at home. Even if you can’t spend the time you want in an aspect of your life be the best at it when you are in it.”

## Conflict of Interest Statement

The author declares that the research was conducted in the absence of any commercial or financial relationships that could be construed as a potential conflict of interest.
